# Supplementary material for: ‘I don’t want anyone to know’: Experiences of obtaining access to HIV testing by Eastern European, non-European Union sex workers in Amsterdam, the Netherlands
Source: PLoS One. 2020 Jul 7;15(7):e0234551. doi: 10.1371/journal.pone.0234551 (PMC7340317; doi:10.1371/journal.pone.0234551)
Supplement: S1 Appendix — (DOCX) [file pone.0234551.s001.docx]

**Annex 1: Data extraction form for analysis of the online advertisements EE migrant FSWs in Amsterdam**

| Number (each advertisement will be assigned with number 01, 02, 03, 04…) |  |
| --- | --- |
| Name of the website |  |
| Web link |  |
| Date of the advertisement |  |
| Name |  |
| Age |  |
| Weight-height or other numerical values provided |  |
| Country of origin (or/and nationality) |  |
| Self-described typology (e.g., escort, companion, etc) |  |
| Business type (in-call, out-call) |  |
| Health communication: medical certificates mentioned or required from client |  |
| Health communication: other |  |
| Safety communication: restrictions mentioned |  |
| Safety communication: location of work |  |
| Safety communication: willingness to travel |  |
| Business communication: service listed |  |
| Business communication: service fees |  |
| Business communication: booking process |  |
| Business communication: the stated type of contract required for booking |  |
| Business communication: payment process (methods and currency) |  |
