# Supplementary material for: ‘I don’t want anyone to know’: Experiences of obtaining access to HIV testing by Eastern European, non-European Union sex workers in Amsterdam, the Netherlands
Source: PLoS One. 2020 Jul 7;15(7):e0234551. doi: 10.1371/journal.pone.0234551 (PMC7340317; doi:10.1371/journal.pone.0234551)
Supplement: S2 Appendix — (DOCX) [file pone.0234551.s002.docx]

**Annex 2: Interview guide for key stakeholders (Phase 1)**

*Thank you very much for agreeing to take part in this interview. You have been approached to participate, because I think that your personal experiences with regard to the vulnerabilities of sex workers and/or migrants in Amsterdam are valuable and important to understand. Also, your experiences with the things that might make it easy or difficult for this vulnerable group to test for HIV are important to recognize. Finally, the insight you might provide to this study may contribute to the understanding of what might hinder migrant female sex workers to test for HIV, and how the uptake of HIV tests might be enhanced among this vulnerable group.*

*To begin with, could you please tell me a bit more about the work you do?*

**Policies and laws**

- Could you tell me about how the undocumented/illegal status of this vulnerable group might influence their access to HIV testing services?
  - Should this be changed according to you?

**HIV testing services**

- Could you tell me about how HIV testing services are offered to this vulnerable group?
  - What do you think about this?
- In what way are migrant female sex workers being informed about an HIV test?
  - What do you think about this?
- In case a migrant female sex worker has been tested positive for HIV, how is this person being referred?
  - What do you think about this?

**Healthcare provider**

- What do healthcare providers think about offering an HIV test to migrant female sex workers?
  - Is there anything that might make it easy for them to offer an HIV test?
  - Is there anything that might make it difficult for them to offer an HIV test?
  - How do they look at the importance of offering an HIV test to this group?

**Migrant female sex workers**

- What do migrant female sex workers think about HIV testing?
  - Is there anything that might make it easy for them to undertake HIV testing?
  - Is there anything that might make it difficult for them to undertake HIV testing?
  - What do they think about the importance to undertake HIV testing annually?
  - How should an HIV test be offered according to migrant female sex workers?
- Do you think that people in their social network might have an influence on whether they test for HIV or not?

**Organizations**

- Could you tell me about some organizations that are involved with sex workers and/or migrants?
- How do these organizations look at the importance of HIV testing among this group?
  - Is there anything that might make it easy for them to pay attention to HIV testing?
  - Is there anything that might make it difficult for them to pay attention to HIV testing?

So I would like to summarize what we have discussed so far. *[Summarise]* Could you tell me what should be changed in order to enhance the uptake of HIV testing services?

Is there anything you would like to add? Do you have any comments or questions left? Would you like to give any advice that you consider as important to this topic?

**Stakeholders**

- Could you tell me which stakeholders should be interviewed as well regarding this topic?

This is the end of the interview. Thank you so much for your time and for participating in our research project!
