# Supplementary material for: ‘I don’t want anyone to know’: Experiences of obtaining access to HIV testing by Eastern European, non-European Union sex workers in Amsterdam, the Netherlands
Source: PLoS One. 2020 Jul 7;15(7):e0234551. doi: 10.1371/journal.pone.0234551 (PMC7340317; doi:10.1371/journal.pone.0234551)
Supplement: S3 Appendix — (DOCX) [file pone.0234551.s003.docx]

**Annex 3: In-depth Interviews with the EE, non-EU migrant FSWs (Phase 2)**

Location:

- To be agreed by the FSWs.

Total time:

- ~1.5-2 hours.

Supplies:

- Audio recording devices.
- Printed consent form (to be filled ONLY if the interview is not recorded).

**Objectives:**

- To identify and analyse context specific vulnerabilities mediating HIV testing and repeated testing in non- EU, EE migrant FSWs and how this is influenced by the different layers of the social ecological model (social networks, intrapersonal and interpersonal levels, you can cite here the few levels that remain).
- To enhance understanding of the perceptions of EE, non-EU migrant FSWs towards different HIV testing modalities.
- To examine previous HIV testing experience at the country of origin and whether this experience influences current perceptions about HIV testing in the destination country.

To examine how health, migration and sex work policies influence perceptions about HIV testing among EE, non-EU migrant FSWs in Amsterdam.

- To identify ideas on how to improve uptake of HIV testing among EE, non-EU migrant FSWs in Amsterdam.

*Figure 1. The approach of the interview: From General to Specific (developed by Tokar A.)*

Life story and migration

Day-to day routine

HIV testing knowledge

Experience of HIV testing

Motivation and barriers

Different HIV testing approaches and modalities

Suggestions on how to improve HIV testing uptake

**Informed Consent and Introductions**

*Thank the participant for her time and introduce yourself.*

**Explain participant that:**

- You are being invited to take part in the research study aiming to understand what would make it easier for women migrated from Eastern Europe to access healthcare services, including testing for HIV in Amsterdam.
- It is important for us to hear about the different experiences, concerns and suggestions of EE female migrants, and who might already undertake HIV testing or plan to do so. We want to learn about your experience in terms of accessing health services, including HIV testing.
- While we aim to help to promote HIV testing uptake among EE female migrants, we cannot guarantee that we will be able to cover all of the needs of participants that may be identified during this study.
- We will not ask you about your HIV status or HIV test result.

Do you have any questions about what I have just explained?

**Informed Consent:**

The interview will take about 2 hours. It will be kept confidential and anonymous within this study. You may use any nick-name you like. Codes will be used for the identification when the data will be kept, analyzed or quoted. We will not record your name anywhere; you are free to use any nick-name you prefer.

We will provide a card (a code) to top up your mobile phone (25 EUR). However, we hope that the information that you share with us, will help to make existing HIV testing modes more sharpen to meet the needs of migrant FSWs. We would like to record digitally our conversation. Only our researchers will hear or have access to the recording.

Do I have permission to record our conversation?

*Turn on the tape recorder if permission is given.*

In this interview I am going to ask you about your experience of accessing health services, including HIV testing. Some of the questions may trigger personal experiences, but you do not need to share that if you are uncomfortable. It is also possible that the discussion might bring up difficult and upsetting issues. You are free to stop the interview at any time or to refuse answering some question. Do you agree to participate in the study and to continue the interview?

**Consent Form** (to be filled ONLY if the interview is not recorded)

In this interview we will talk about your experience of accessing health services, including HIV testing.

The interview will take about 2 hours. It will be kept confidential and anonymous within this study. You may use any nick-name you like. Codes will be used for identification when the data will be kept, analyzed or quoted. We will not record your name anywhere.

In this interview I am going to ask you about your experience of accessing health services, including HIV testing. Some of the questions may trigger personal experiences, but you do not need to share that if you are uncomfortable. It is also possible that the discussion might bring up difficult and upsetting issues. We will provide a card (a code) to top up your mobile phone (25 EUR). You are free to stop the interview at any time or to refuse answering some question. Do you have any questions about what I have just explained?

Do you agree to participate in the interview?

___________________________ ___________________

Signature of participant Date

1. **Introductions and Ice breaking (Life story)**

*Before we begin, I would like to know more about you and your life. Can you tell me about yourself and those events which were important for you?*

***Probes:***

- *I’d like to know how long have you been living in Amsterdam?*
- *How did you migrate here?*
- *Why have you decided to go to Amsterdam?*

1. **Can you please tell me about your day-to day life in Amsterdam?**

- *How do you feel living in the other country?*
- *Please, describe what were you doing yesterday?*
- *Please, describe what do you plan to do tomorrow?*
- *Where do you live in Amsterdam?*
- *Where do you work? Can you describe your working day?*
- *How do you spend your leisure time?*
- *(if it doesn’t emerge) Do you travel back to your home country? How often?*

If it does not emerge, in the end of the section ask about age, education and family status: “Sorry, but I would like to ask you about several issues which are important for the study, but they did not come out during our discussion: your age, education and family status?”

1. **HIV testing knowledge**

*As I’ve already mentioned, we are interested to know your opinions towards HIV testing. Do you know where it is possible to undergo HIV testing in Amsterdam?*

***Probes:***

- *Have you ever received any information on where it is possible to undergo HIV testing in Amsterdam? How and when have you received this information?*
- *How do you know where it is possible to test? Who informed you?*

1. **HIV testing experience**
2. *Have you ever thought to undergo HIV testing? When? Why yes or why not?*
3. *And did you got tested? Can you please describe how it was, I want to know your experience? (****If no, ask if she is planning to test in future? And go to the question C****)*
4. *(Only for those who were tested before) Let’s try go back and to remember your previous experience of HIV testing, including the country of origin.*
5. *I would like to know more about your motivation to test. Why have you decided to test? Ask separately about every test. (For participant, who has never* ***been tested****, ask what can motivate her to test and why?)*

***Probes:***

- *(if it applies) If the participant has never been tested in the country of origin, ask why. If the participant has never been tested in the country of destination, ask why.*
- *What can motivate you to test* ***now****? Can you give me an example?*
- *What can support you?*
- *What can motivate migrant FSW to test? Can you give me an example?*
- *(if it does not emerge) What can motivate people to repeat the test?*
- *What motivated you* ***to repeat test****? Why? How often? (define the term “regularly”)*
- *Why have you decided to* ***repeat*** *test? Why have you decided to test again?*
- *Are there any differences in the country of origin (name it) and in Amsterdam?*

1. *I also would like to know your opinion on the barriers to HIV testing? (For participant, who has* ***never been tested****, ask what was her personal reasoning not to test)*

***Probes:***

- *Why do you think so?*
- *Can you give me an example?*
- *Were there any barriers to access HIV testing for you? Can you give me an example?*
- *Why might you decide not to test now? Why?*
- *(if it doesn’t emerge) Ask about illegal status of migrants and illegal employment, drug use, HIV testing policies (ART).*

***(if it does*** *not emerge) Are there any differences in HIV testing in the country of origin (name it) and in Amsterdam?*

- *(if it does not emerge) Are there any barriers to* ***repeat*** *test regularly?*

1. *Now considering mentioned, let’s talk about HIV testing approaches, which were applied in each case. Can you tell me how were you tested?* ***If participant has never been tested,*** *ask did she heard about different HIV testing approaches (let her recall what she knew and ask probes if it applies).*

***Probes:***

- *Can you tell me where (places) were you tested?*
- *Can you try to remember if you were tested at hospital (venous blood/ finger blood)? Which health facility was it: TB clinic, STI clinic, reproductive settings, GP, emergency department?*
- *Can you try to recall if you were tested at the mobile unit?*
- *Can you try to recall if you were tested at the non-governmental organization/community centre/shelter?*
- *Can you try to remember if you were tested at pharmacy?*
- *Did you test yourself? Did you buy from pharmacy HIV test?*
- *Can you try to remember if you were tested with your friend/spouse/ or group of other people?*

1. *What did you like about the approach? (****If participant has never been tested:*** *what are the pros of the approach/es she mentioned by participant? Why so?).*

***Probes:***  *Anything else? Can you give me an example?*

1. *What didn’t you like about the approach? (****If participant has never been tested:*** *what are the cons of the approach/es she mentioned by participant? Why so?).*

***Probes:*** *Anything else? Can you give me an example?*

*If participant will not mention different dimensions of HIV testing, a****sk about each testing experience.***

1. **Suggestions on how to increase HIV testing uptake**
2. *On the base of the information which you’ve reported previously, I can conclude that it’s important for you to… (paraphrase) Am I right? Anything else*
3. *What would you suggest to increase HIV testing uptake in migrant FSWs?*

***Probe:***

- *What would you suggest to improve HIV testing approaches (refer to the personal experience)? Why that?*
- *Have you seen/participated in the campaigns promoting HIV testing?*
- *What were those? Or Can you describe them in details?*
- *Can you describe your participation in the details?*
- *What do you think of such activities, why?*
- *Which is the role of Internet/media to promote HIV testing, why?*

1. **WRAPPING UP**

Is *there* anything else that you’d like to discuss today about HIV testing in migrant EE, non-EU FSWs? Are *there* any questions that you’d like to ask me about anything that we have discussed today?

1. **Conclusion**

*Thank you for your time and cooperation. I’d also like to remind you that we would be disseminating the information from the study during one of our group activities with the key stakeholders. You are most welcome to attend (give the contact tel. number and email address).*
