# Supplementary material for: ‘I don’t want anyone to know’: Experiences of obtaining access to HIV testing by Eastern European, non-European Union sex workers in Amsterdam, the Netherlands
Source: PLoS One. 2020 Jul 7;15(7):e0234551. doi: 10.1371/journal.pone.0234551 (PMC7340317; doi:10.1371/journal.pone.0234551)
Supplement: S4 Appendix — (DOCX) [file pone.0234551.s004.docx]

**Annex 3: In-depth Interviews with stakeholders (Phase 3)**

Location:

- To be agreed by the participant.

Total time:

- ~1.5

Supplies:

- Audio recording devices.
- Printed consent form (to be filled ONLY if the interview is not recorded).

**Objectives:**

- Examine stakeholders opinions on: (a) context specific vulnerabilities mediating HIV testing and repeated testing in non- EU, EE migrant FSWs and how this is influenced by the different layers of the social ecological model (social networks, intrapersonal and interpersonal levels, you can cite here the few levels that remain) (b) different HIV testing modalities and possibility to apply them in the group of non- EU, EE migrant FSWs (c) how health, migration and sex work policies influence HIV testing uptake among EE, non-EU migrant FSWs in Amsterdam (including a new law on sex work).
- To identify ideas on how to improve uptake of HIV testing among non-EE, EU migrant FSWs in Amsterdam.

*The approach of the interview: From General to Specific (developed by Tokar A.)*

Introductions and ice breaker

Migration patterns

Knowledge and Experience of HIV testing

Motivation and barriers

Different HIV testing approaches and modalities

Suggestions on how to improve HIV testing uptake

**Informed Consent and Introductions**

*Thank the participant for her time and introduce yourself.*

**Explain participant that:**

- You are being invited to take part in the research study aiming to understand what would make it easier for women migrated from Eastern Europe to access healthcare services, including testing for HIV in Amsterdam.
- It is important for us to hear about the different experiences, concerns and suggestions of stakeholders on how to improve HIV testing in EE female migrants.
- While we aim to help to promote HIV testing uptake among EE female migrants, we cannot guarantee that we will be able to cover all of the needs of participants that may be identified during this study.
- We will not ask you about your HIV status or HIV test result.

Do you have any questions about what I have just explained?

**Informed Consent:**

The interview will take about 1.5-2 hours. It will be kept confidential and anonymous within this study. You may use any nick-name you like. Codes will be used for the identification when the data will be kept, analyzed or quoted. We will not record your name anywhere; you are free to use any nick-name you prefer. You will not receive any direct benefit from taking part in this study. However we hope that the information that you share with us, will help to make existing HIV testing modes more sharpen to meet the needs of migrant FSWs. We would like to record digitally our conversation. Only our researchers will hear or have access to the recording. Do I have permission to record our conversation?

*Turn on the tape recorder if permission is given.*

In this interview I am going to ask you about your experience of accessing health services, including HIV testing. Some of the questions may trigger personal experiences, but you do not need to share that if you are uncomfortable. It is also possible that the discussion might bring up difficult and upsetting issues. You are free to stop the interview at any time or to refuse answering some question. Do you agree to participate in the study and to continue the interview?

**Consent Form** (to be filled ONLY if the interview is not recorded)

In this interview we will talk about your experience of accessing health services, including HIV testing. The interview will take about 1-2 hours. It will be kept confidential and anonymous within this study. You may use any nick-name you like. Codes will be used for identification when the data will be kept, analyzed or quoted. We will not record your name anywhere. In this interview I am going to ask you about your experience of accessing health services, including HIV testing. Some of the questions may trigger personal experiences, but you do not need to share that if you are uncomfortable. It is also possible that the discussion might bring up difficult and upsetting issues. You are free to stop the interview at any time or to refuse answering some question. Do you have any questions about what I have just explained? Do you agree to participate in the interview?

___________________________ ___________________

Signature of participant Date

**Introductions and Ice breaking**

*Before we begin, I would like to know more about your work. Can you tell me about the work you do on the day-to-day basis?*

***Probes:***

- *(if it doesn’t emerge) I’d like to know if your work is related to migrants/sex workers? Can you tell in details?*
- *(if it doesn’t emerge) Do you work with migrant sex workers? Can you give an example?*
- *(if it doesn’t emerge) Do you work with illegal/legal migrant SWs? How?*
- *(if it doesn’t emerge) Do you work with EE migrant SWs?*

1. **Can you please tell me about migration patterns in Amsterdam (AMS)/ the Netherlands (NL)?**
2. *What do you think about migration flows in AMS/NL? Can you give some statistics? Can you give some examples?*
3. *What do you think about illegal migration? Can you give some statistics? Can you give some examples?*
4. *And if to talk about migrant in sex work?*
5. *What do you think about EE migration?*
6. *And EE migrant FSWs? Can you illustrate with an example?*

- ***Probes:***
- *From where do people migrate to AMS/NL? (ask about main counties of origin)*
- *Why FSWs from EE migrate to AMS/NL? (ask about circular migration-migration in between EU countries and back to the country of origin)*
- *(if it doesn’t emerge) What are the regulations for migration of FSWs (legal vs illegal)? What do you think of those? (ask about new law on sex work and how it will change migration regulations)*

In the end of this section ask about socio-demographics of EE female sex workers (age/education/marital status).

1. **HIV and knowledge and testing experience**
2. *What do you think about access to health care for migrant FSWs (legal vs illegal)?*
3. *Can you tell me in your own words why one should test (risk behavior)? Why so? Do migrant need to be tested? And sex workers?*
4. *What do you think about EE migrant FSWs and access to health? Why so? Do they need to be tested for HIV? Whys so?*

***Probes:***

- *How can they receive any information on where it is possible to undergo HIV testing in Amsterdam?*
- *Are there any differences in HIV testing policies in the EE countries and in Amsterdam, NL? Can you tell me more about HIV testing policies in AMS? What do you think about them?*
- *How and when this information is spread?*
- *And did they get tested for HIV? Can you please describe how it happens?*

1. *Let’s try to go back and try to think about repeated testing? How often have they been tested? Do you know if they test in the home country?*

***!!! define the term “regularly”!!!!***

1. *Now, I would like to know more about motivation to test. Why do EE FSWs agree to test or don’t? Can you give examples?*

***Probes:***

- *What can motivate migrant FSW to test? Can you give me an example? What can support them?*
- *(if it does not emerge) What can motivate people to repeat the test? Why?*
- *What can be improved? How?*
- *(if it does not emerge) And if to talk about the new law on sex work? What do you think of it in terms of motivation to test?*

1. *I also would like to know your opinion on the barriers to HIV testing? Why do you think so? Can you give me an example?*

***Probes:***

- *Were there any barriers to access HIV testing for migrants/SWs/migrant FSWs/EE migrant FSWs? Can you give me an example?*
- *Why migrant FSWs decide not to test* ***now****? Why?*
- *Ask about illegal status of migrants and illegal employment, drug use, HIV testing policies (****ART!****).*
- *Are there any barriers to* ***repeat*** *test?*
- *And if to talk about the new law on sex work? What do you think of it in terms of motivation to test?*
- *Now considering mentioned above, let’s talk about HIV testing approaches, which were applied or might be applied. Can you tell me how migrant FSWs have been tested?*
- *Can you tell me where (places) migrant FSWs (legal vs illegal) can be tested?*

1. *What do you like about the approach you have named? Why so? -refer to each approach which was named by the respondent)? And what do you think about other approaches? Why so? Anything else? Can you give me an example?*

***Probes:***

- *And what about counselling during testing?*
- *Please, describe how participants agree to test? Do they sign any forms or gave the verbal consent?*
- *How results are communicated to migrant FSWs?*
- *Do they collect test results? How? How are they reported?*
- *Can migrant FSWs access ART? Can you give an example?*

1. **Suggestions on how to increase HIV testing uptake**
2. *On the base of the information which you’ve reported previously, I can conclude that it’s important for you to… (paraphrase) Am I right? Anything else*
3. *What would you suggest to increase HIV testing uptake in migrant FSWs?*

***Probes:***

- *What would you suggest to improve HIV testing approaches (refer to mentioned above)? Why that?*
- *Were there any campaigns promoting HIV testing? What were those? Can you describe them in details?*
- *What do you think of such activities, why?*
- *Which is the role of Internet/media to promote HIV testing, why?*

1. **WRAPPING UP**

Is *there* anything else that you’d like to discuss today about HIV testing in migrant EE, non-EU FSWs? Are *there* any questions that you’d like to ask me about anything that we have discussed today?

1. **Conclusion**

*Thank you for your time and cooperation. I’d also like to remind you that we would be disseminating the information from the study during one of our group activities with the key stakeholders. You are most welcome to attend (give the contact tel. number and email address).*
