# Supplementary material for: ‘I don’t want anyone to know’: Experiences of obtaining access to HIV testing by Eastern European, non-European Union sex workers in Amsterdam, the Netherlands
Source: PLoS One. 2020 Jul 7;15(7):e0234551. doi: 10.1371/journal.pone.0234551 (PMC7340317; doi:10.1371/journal.pone.0234551)
Supplement: S5 Appendix — (DOCX) [file pone.0234551.s005.docx]

| **Annex 5: Coding Framework** | | | |
| --- | --- | --- | --- |
| **N** | **Code** | **Code Network** | **Code Group** |
| 1 | Macro_Healthcare funding_formal and informal insurance payments (35) | (1)Healthcare funding | **Macro** |
| 2 | Macro_Healthcare funding_free ART_test (33) | (1)Healthcare funding | **Macro** |
| 3 | Macro_Healthcare funding_healthcare entitlements (7) | (1)Healthcare funding | **Macro** |
| 4 | Macro_HIV testing policies forced (1) | (2)HIV testing policies | **Macro** |
| 5 | Macro_HIV testing policies_delivery (15) | (2)HIV testing policies | **Macro** |
| 7 | Macro_HIV testing policies_lack of confidentiality (7) | (2)HIV testing policies | **Macro** |
| 8 | Macro_HIV testing policies_mandatory (8) | (2)HIV testing policies | **Macro** |
| 9 | Macro_HIV testing policies_non-anonymous (6) | (2)HIV testing policies | **Macro** |
| 10 | Macro_Migration policies (72) | (3)Migration policies | **Macro** |
| 11 | Macro_Sex work policies_employment_officially (86) | (4)Sex work policies | **Macro** |
| 12 | Macro_SW policies_Criminalization of illegal/unregistered sex work (25) | (4)Sex work policies | **Macro** |
| 13 | Macro_SW policies_Trafficking policies (59) | (4)Sex work policies | **Macro** |
| 14 | Macro_Criminalization_of sex work (shrinking sex work in AMS)(21) | (4)Sex work policies | **Macro** |
| 15 | Meso_Costs_density of HIV testing sites (13) | (1)Costs | **Meso** |
| 16 | Meso_Costs_time constraints (17) | (1)Costs | **Meso** |
| 17 | Meso_Costs_travel costs to collect test results (2) | (1)Costs | **Meso** |
| 18 | Meso_Cultural mediator (5) | (2)Organizations/networks working with sex workers | **Meso** |
| 19 | Meso_NGOs_healthcare_workers (65) | (2)Organizations/networks working with sex workers | **Meso** |
| 20 | Meso_Institutional challenges | (2)Organizations/networks working with sex workers | **Meso** |
| 21 | Meso_Special services | (2)Organizations/networks working with sex workers | **Meso** |
| 22 | Meso_Police (33) | (2)Organizations/networks working with sex workers | **Meso** |
| 23 | Meso_Self-organizations (19) | (2)Organizations/networks working with sex workers | **Meso** |
| 24 | Meso_Social network_ peers (24) | (3)Social network | **Meso** |
| 25 | Meso_Social network_family (4) | (3)Social network | **Meso** |
| 26 | Meso_Socil network_ managers/pimps (11) | (3)Social network | **Meso** |
| 27 | Meso_ Stigma & discrimination _discrimination from healthcare providers (13) | (4)Stigma & discrimination | **Meso** |
| 28 | Meso_ Stigma & discrimination _discrimination of family members (3) | (4) Stigma & discrimination | **Meso** |
| 29 | Meso_Stigma&discrimination_fear of HIV positive result (2) | (4) Stigma & discrimination | **Meso** |
| 30 | Meso_Stigma&discrimination_fear of illegal status/ if testing HIV+, fear of deportation, visa cancellation) (13) | (4) Stigma & discrimination | **Meso** |
| 31 | Meso_ Stigma & discrimination _fear to disclose history of SW/drug use (27) | (4) Stigma & discrimination | **Meso** |
| 32 | Meso_Stigma & discrimination_ _negative perception of the quality of healthcare (6) | (4) Stigma & discrimination | **Meso** |
| 33 | Meso_ Stigma & discrimination _self-stigmatization (7) | (4) Stigma & discrimination | **Meso** |
| 34 | Meso_Anti-migrants views (12) | (4) Stigma & discrimination | **Meso** |
| 35 | Meso_SW policy delivery_victimization (13) | (4) Stigma & discrimination | **Meso** |
| 36 | Meso_SW venue_indoors (21) | (5) Sex work venue | **Meso** |
| 37 | Meso_SW_venue_escort via Internet (73) | (5) Sex work venue | **Meso** |
| 38 | Meso_SW_venue_outdoors (15) | (5) Sex work venue | **Meso** |
| 39 | Meso_Income level of SW venue (2) | (5) Sex work venue | **Meso** |
| 40 | Meso_Trust (26) | (6)Trust | **Meso** |
| 41 | Micro_HIV knowledge (where, how, local policies) (29) | (1) Knowledge | **Micro** |
| 42 | Micro_Risk_awareness (perceived risk, perception towards HIV prevalence) (25) | (2)Risk awareness | **Micro** |
| 43 | Micro_risk_beh_age at first sex (1) | (3)Risk behaviour | **Micro** |
| 44 | Micro_risk_beh_condom use (5) | (4)Risk behaviour | **Micro** |
| 45 | Micro_risk_beh_drug and alcoh use (4) | (4)Risk behaviour | **Micro** |
| 46 | Micro_risk_beh_type and N of sexual partners (6) | (4)Risk behaviour | **Micro** |
| 47 | Micro_socio_dem_self defeniton of sex work (19) | (5)Socio-demographic information | **Micro** |
| 48 | Micro_socio-dem_age (9) | (5)Socio-demographic information | **Micro** |
| 49 | Micro_socio-dem_background experience_country of origin (24) | (5)Socio-demographic information | **Micro** |
| 50 | Micro_socio-dem_education (6) | (5)Socio-demographic information | **Micro** |
| 51 | Micro_socio-dem_family status (5) | (5)Socio-demographic information | **Micro** |
| 52 | Micro_socio-dem_income (15) | (5)Socio-demographic information | **Micro** |
| 53 | Micro_socio-dem_knowing local laws (15) | (5)Socio-demographic information | **Micro** |
| 54 | Micro_Frequent travel (3) | (5)Socio-demographic information | **Micro** |
| 55 | Micro_socio-dem_language (34) | (5)Socio-demographic information | **Micro** |
| 56 | How to improve_HIV testing (88) | **Solutions how to improve** | |
