# Supplementary material for: ‘I don’t want anyone to know’: Experiences of obtaining access to HIV testing by Eastern European, non-European Union sex workers in Amsterdam, the Netherlands
Source: PLoS One. 2020 Jul 7;15(7):e0234551. doi: 10.1371/journal.pone.0234551 (PMC7340317; doi:10.1371/journal.pone.0234551)
Supplement: Attachment — Submitted filename: Response to reviewer_Tokar.docx [file pone.0234551.s006.docx]

February 24, 2020

PLOS ONE

Dear all

On behalf of my co-authors (J. Osborne, R. Hengeveld, Jeffrey V. Lazarus, Jacqueline E.W. Broerse) I am re- submitting our manuscript entitled “I don’t want anyone to know’: experiences of obtaining access to HIV testing by Eastern European non-European Union sex workers in Amsterdam” (*Manuscript ID: PONE-D-19-24622*). Below, we provide a response to each point raised by the reviewer(s).

| **Review Comments to the Author** | |
| --- | --- |
| **Comments** | **Responses** |
| 1. Recommend "sex workers who use drugs" rather than "drug-using sex workers." | Thank you very much for the comment. We have changed "drug-using sex workers" to "sex workers who use drugs".  (Introduction Section- Page 3) |
| 2. Check wording please: "...problems of being [able] to work legally..." - consider more direct wording. Rather than problems of being able to, consider "...together with being unable to work legally..." | We have changed "...problems of being [able] to work legally... " to "...together with being unable to work legally..."  (Introduction Section -Page 4) |
| 3. Thank you for highlighting the limited numbers in phase 2 - migrant sex workers, particularly those working without legal status, are absent from data collection. Could this be expanded? I think it's a methodological note worth explaining - so that future research on this area/this population can learn from the authors' experiences. What would they have done differently if they could re-design their strategy to access this population? | Thank you very much for the comment.  We have written a separate methodological paper focusing on different methodological issues, challenges as well as presenting some reflections. This paper is now under review. We have added:  “A more detailed descriptions and reflections on methodological aspects of this study, including sampling, data collection and ethical issues, could be found in another article, which we have written.”  (Methods Section- Page 13) |
| 4. Should phase 3, interviewee number 6 be re-categorized to phase 2? | Interviewee number six, was not a migrant from Eastern Europe. This was a Dutch resident, who was born in the Netherlands and at the moment of our interview, worked as a street-based sex worker. Also, this person was an activist and a member of one sex worker-led organization in the Netherlands. |
| 5. Is there a reason that only one interview tools was included in the submission? "Interview guide for key stakeholders" - is this from phase 1 or phase 3? | The initially included guide was from Phase 1.  We have included now all the guides as annexes.  (Annex 2-4 - Pages 35-44) |
| 6. Were the selected criteria for "key stakeholders" in phases 1 and 3 different? It seems like the same pool of stakeholders? | The pool of respondent for Phase 3 was defined based on the results of Phases 1 and 2. It included some of the NGOs, which we have contacted in the Phase 1 (three of them), yet we have never talked twice to the same person. Moreover, as you may see from the list of participants (Table 1), in Phase 1 a broad range of stakeholders was included (e.g., journalists), while in Phase 2 we were able to focus exclusively on those stakeholders working with EE non-EU migrant FSWs. |
| 7. To readers less familiar with qualitative methods, please ensure the difference between semi-structured interview and in-depth interview and the reasons to select one method or the other are clear | Thank you very much for the comment. We have added a description of both methods as well as reasons why these methods have been applied. Terms “semi-structured interviews” and “in-depth, narrative interviews” were used in accordance with definitions given by *Judith Green and Nicki Thorogood, “Qualitative methods for Health Research”, SAGE Ltd, 2004.*  “Since we aimed to collect information on predefined topics in this first phase of the project, we conducted semi-structured interviews, in which a researcher set the agenda in terms of topics covered, but the respondent determined the kinds of information obtained as well as the relative importance of this information.” (Methods Section – Pages 8-9)  “In Phase 2 we used in-depth, narrative interviews as this method allowed respondents to have enough time to develop their own accounts of the issues important to them, while interviewer served rather as a facilitator of this process. Respondents determined the conversation flow.”  (Methods Section – Pages 9) |
| 8.Wording check: "...and use their power [to] get extra sexual services for free | Thank you very much for the comment.  We have changed “and use their power to get extra sexual services for free.”  (Results Section– Page 15) |

Thank you very much for your consideration of this manuscript.

Sincerely,

Anna Tokar

Barcelona Institute for Global Health (ISGlobal),

Hospital Clínic – University of Barcelona

Rosselló, 132, 4th

ES-08036, Barcelona, Spain

E-mail: [anna.tokar@isglobal.org](mailto:anna.tokar@isglobal.org)
